# Research Progress on Inactivation of Bacteriophages by Visible-Light Photocatalytic Composite Materials: A Mini Review

**DOI:** 10.3390/ma17010044

**Published:** 2023-12-21

**Authors:** Deqiang Zhao, Heng Lu, Qingkong Cheng, Qi Huang, Jing Ai, Zhibo Zhang, Hainan Liu, Zongfei He, Qiuhong Li

**Affiliations:** 1 School of River and Ocean Engineering, Chongqing Jiaotong University, Chongqing 400074, China; luheng0723@163.com (H.L.); huangqi0903@163.com (Q.H.); hainanliu1998@163.com (H.L.); liqiuhong023@163.com (Q.L.); 2 National Engineering Research Center for Inland Waterway Regulation, Key Laboratory of Hydraulic and Waterway Engineering of the Ministry of Education, Chongqing 400074, China; 3 Department of Materials and Environmental Chemistry, Stockholm University, SE-106 91 Stockholm, Sweden; jing.ai@mmk.su.se (J.A.); zhibo.zhang@mmk.su.se (Z.Z.); 4 Joint Graduate Training Base for Resources and Environment between Chongqing Jiaotong University and Chongqing Gangli Environmental Protection Co., Ltd., Chongqing Jiaotong University, Chongqing 400074, China; 5 School of Environmental and Municipal Engineering, Lanzhou Jiaotong University, Lanzhou 730070, China; 18723471663@163.com

**Keywords:** composite materials, visible-light photocatalyst, bacteriophage, disinfection, water treatment

## Abstract

Infectious diseases caused by waterborne viruses have attracted researchers’ great attention. To ensure a safe water environment, it is important to advance water treatment and disinfection technology. Photocatalytic technology offers an efficient and practical approach for achieving this goal. This paper reviews the latest studies on visible-light composite catalysts for bacteriophage inactivation, with a main focus on three distinct categories: modified UV materials, direct visible-light materials and carbon-based materials. This review gives an insight into the progress in photocatalytic material development and offers a promising solution for bacteriophage inactivation.

## 1. Introduction

Waterborne viruses in drinking water and infectious diseases caused by waterborne viruses have gradually attracted people’s attention [1]. In order to ensure the safety of drinking water, it is of great significance to develop an efficient and low-cost water treatment and disinfection method. Phages are commonly found in sewage and polluted water, and their morphology and structure are similar to those of enteroviruses [2]. Single-stranded RNA phages are thus expected to replace some enteroviruses (such as *poliovirus PV-1*) as virus indicators for sewage evaluation. With *Escherichia coli* serving as the specific host, phages are safe as virus indicators. Additionally, phages possess clear nucleic acid sequences and simple structures, and provide for a short experimental period, ease of operation, and low cost with objective evaluation results, aligning with international standard methods suitable for assessing and testing water quality. As a result, bacteriophage MS2 has been recommended by the US EPA as an indicator organism for viruses in water and is now widely used for evaluating water and sewage disinfection efficiency and understanding the mechanism of virus inactivation [3].

Chemical disinfectants commonly used in traditional water treatment include chlorine, chlorine dioxide and ozone. However, studies have found that the use of chemical disinfection processes will form a number of harmful compounds (such as formaldehyde and acetaldehyde produced by using ozone) and toxic disinfection byproducts (DBPs), seriously threatening human health [4,5]. Ultraviolet light (UV) disinfection and filtration technology are also widely used water treatment technologies. Compared with traditional chemical disinfectants, ultraviolet disinfection is more effective without the production of DBPs. However, the biggest disadvantage of ultraviolet disinfection is the lack of continuous disinfection ability, and thus the ultraviolet method is only suitable for primary disinfection [6]. Filtration technology is used to separate pollutants in water using physical barriers, effectively removing particles, microorganisms and pollutants in water. However, filtration technology has high operating costs, restricting its wide application [7]. Given the virus’ small size, unique structure and high pathogenicity, it remains a challenging task to effectively eliminate waterborne viruses using current disinfection technology. Therefore, an efficient and environmentally friendly alternative disinfection method is in demand.

As a new advanced oxidation technology, photocatalysis has attracted extensive attention. Photocatalysis is a versatile technology used to treat heavy metals [8], eliminate organic pollutants [9,10,11,12], and reduce nitrates [13]. Via the photoexcitation of electrons on catalyst surfaces, it facilitates the reduction or oxidation reactions of heavy metal ions, transforming them into stable or precipitated forms, thereby diminishing the toxicity of heavy metals in water or soil. By leveraging the photosensitivity of catalysts to generate active oxides, organic substances undergo oxidation and degradation into water and carbon dioxide, mitigating the organic load in water or soil [14,15,16,17]. In contrast to conventional disinfection methods, photocatalytic disinfection showcases extensive bactericidal effectiveness [18], simultaneously addressing a variety of microorganisms such as bacteria, viruses, and fungi. The notable advantage lies in the lack of chemical residues after treatment, minimizing adverse impacts on both the environment and human health [19]. Moreover, the sustainability of the photocatalytic disinfection process is noteworthy, as it only requires a light source to activate the catalyst, eliminating the necessity for frequent chemical replacements or additions. It uses solar energy directly to drive chemical reactions, facilitating the decomposition of organic substances, thus surpassing the limitations of traditional disinfection methods.

In the photocatalytic process, when light irradiates the photocatalyst with the energy of the radiation equal to or greater than the electron band gap, the surface of the photocatalyst generates electron-hole (e^−^-h^+^) pairs, and those pairs then separate and migrate to the photocatalyst surface to participate in redox reactions [20]. For instance, photogenerated e^−^ reduces O_2_ in water to •O_2_^−^, while the photogenerated h^+^ oxidizes the surface H_2_O/OH^−^ to •OH. Both •O_2_^−^ and •OH are reactive oxygen species (ROSs), which are highly active oxidants that play an important role in the process of the photocatalytic inactivation of viruses [5].

TiO_2_ is one of the most common photocatalysts, used as a fungicide for the first time in 1985, successfully inactivating photocatalytic *Saccharomyces cerevisiae* under UV irradiation [21]. TiO_2_ inactivates microorganisms by absorbing UV to produce ROSs, and it is widely used in water disinfection due to its low cost, high disinfection efficiency, non-toxicity and good light stability [22,23]. In addition to TiO_2_, many materials have also been used in the photocatalytic degradation of refractory organics, water disinfection and other fields, such as ZnO, WO_3_, and g-C_3_N_4_ [24,25,26]. In recent years, some studies have reported good performance of photocatalyst materials for the removal of *Salmonella*, *Pseudomonas aeruginosa* and viruses such as MS2 phage and ΩX174 phage [27,28]. In spite of the success of UV photocatalytic disinfection [29,30,31,32], solar-induced photocatalytic water disinfection still faces challenges. At present, most semiconductor materials can only achieve light response in the ultraviolet region, which only accounts for 4–5% of the entire solar spectrum, while the visible light accounts for about 44%. Accordingly, how to effectively utilize visible light in photocatalytic disinfection is an urgent problem to be solved, and the development of visible-light-active photocatalysts has become a research hotspot in the development of photocatalytic disinfection.

With continuous research and development, many visible-light photocatalytic composite materials have been developed using metal doping and semiconductor composite methods. Photocatalytic activity may be enhanced by doping TiO_2_ with non-metals or/and noble and transition metals, which significantly influence the photo-reactivity, charge carrier recombination rates and interfacial electron-transfer rates. These composite catalysts have been shown to have superior antiviral and antibacterial behavior compared to pure titania [33]. They also showcase significant potential in hydrolyzing and degrading organic pollutants [34,35,36]. Despite these advancements, the practical application of photocatalytic water disinfection technology faces challenges associated with the immobilization of photocatalysts and the design of photocatalytic reactors, mainly attributed to the prevalent use of powder-form photocatalysts [5]. Future research endeavors will be directed towards the development of more efficient and easily recyclable photocatalysts, including metal–organic frameworks (MOFs) with semiconductor-like properties [37] and photocatalyst with magnetic properties [38]. These advanced photocatalysts possess notable advantages, including the presence of open metal sites and catalytically active ligands, which empower them to act as charge carriers upon photoexcitation. Their exceptional design flexibility arises from the ease of modifying metal centers or organic ligands, distinguishing them significantly from conventional semiconductors [39].

This review aims to introduce the latest research progress of visible-light composite photocatalysts for bacteriophage inactivation and focuses on the synthesis and modification of visible-light catalysts. The bacteriophage inactivation mechanism and efficiency of several visible-light photocatalysts are also reviewed with perspectives on the development of visible-light photocatalytic disinfection.

## 2. Modified Ultraviolet-Response Composite Materials

The most widely used photocatalytic materials including ZnO and TiO_2_ are wide band gap semiconductors that can only be activated in the ultraviolet region and cannot directly absorb visible light. To achieve photo-responsiveness in the visible region, photocatalytic materials have been modified and developed using different methods.

### 2.1. Cu-TiO_2_

**Figure 1:**
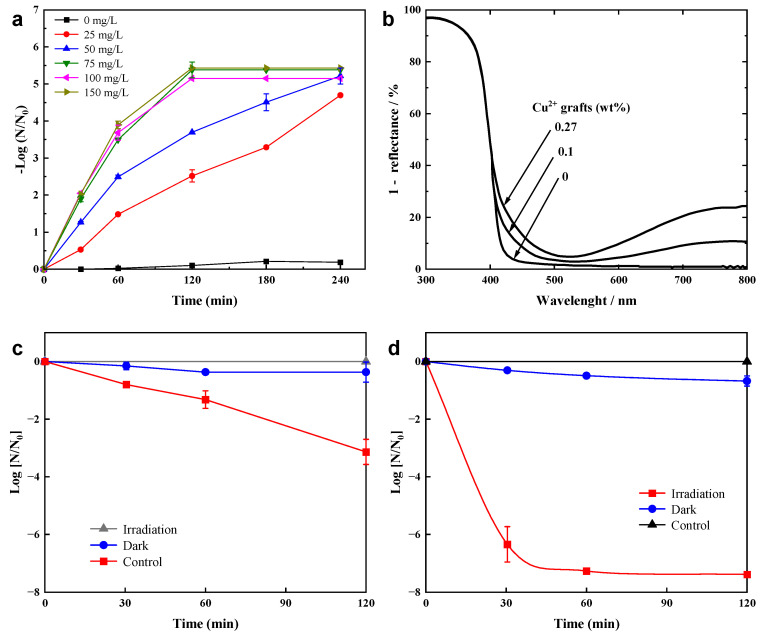
(**a**) Photocatalytic disinfection of bacteriophage f2 using different catalyst dosages. Visible-light intensity: 100 mW/cm^2^, initial virus concentration: 10^5^ PFU/mL, pH = 7, temperature: 25 °C. Reproduced from [40], Environmental Pollution, with permission from Elsevier Ltd. Press 2018. (**b**) UV−vis diffuse reflectance spectra of rutile TiO_2_ and Cu (II)/TiO_2_. Reproduced with permission from [42]. Copyright 2009 American Chemical Society. Inactivation of bacteriophage Qβ by photocatalyst under dark conditions and visible-light irradiation. (**c**) Cu(II)-TiO_2_ (pH = 7), (**d**) Cu(II)-TiO_2_ (pH = 12). Reproduced from [43], Journal of Materials Chemistry A, with permission from The Royal Society of Chemistry Press 2015.

In the photocatalytic process, a major limitation of TiO_2_ is its large band gap, which can only be activated by light in the near-ultraviolet region. Also, the recombination of photogenerated charge carriers of TiO_2_ reduces the overall quantum efficiency. The above problems can be solved by metal doping (such as Cu and Ag). Zheng et al. [40] synthesized Cu-TiO_2_ nanofiber photocatalysts using electrospinning, which confirmed efficient disinfection performance for bacteriophage f2 under visible-light irradiation and this performance was not affected by reaction pH. The removal efficiency of phage f2 by Cu-TiO_2_ remained high even when the pH was close to the point of zero charge (PZC) (pH = 6, 7 was neutral). Under visible-light irradiation, the removal efficiency increased with the increase in visible-light intensity, catalyst dosage and temperature, but decreased with increasing initial virus concentration. With a temperature of 25 °C, pH = 7 and a concentration of 75 mg/L Cu-TiO_2_ nanofibers, the virus with an initial concentration of 10^5^ PFU/mL was completely inactivated after 240 min of visible-light irradiation, showing the best disinfection performance (Figure 1a). Irie et al. [41] created a Cu(II)-TiO_2_ visible-light photocatalytic material by grafting Cu(II) nanoclusters onto TiO_2_. As can be seen from Figure 1b, visible-light absorption improved in the 410 to 450 nm rangeand over 650 nm for Cu(II)-TiO_2_. The absorption of 410–450 nm is due to the fact that the e^−^ in the valence band of TiO_2_ are excited into Cu(II) under visible-light irradiation via an interfacial charge transfer process [42], while the absorption at over 650 nm is ascribed to the d-d transition of Cu(II). Liu et al. [43] optimized the synthesis of Cu(II)-TiO_2_ nanocomposites and prepared thin film samples by coating a Cu(II)-TiO_2_ suspension onto glass substrates. As shown in Figure 1c,d, the highest photocatalytic activity of the Cu(II)-TiO_2_ film showed at a pH of 12. After 30 min of visible-light irradiation, the bacteriophage decreased by 6.5log (Figure 1d), and the virus inactivation reached 99% and 99.99% after 1 h and 2 h of visible-light irradiation, respectively. The experimental data show that Cu(II)-TiO_2_ has efficient inactivation efficiency on bacteriophage Qβ under visible-light irradiation.

### 2.2. Cu_x_O-TiO_2_

**Figure 2:**
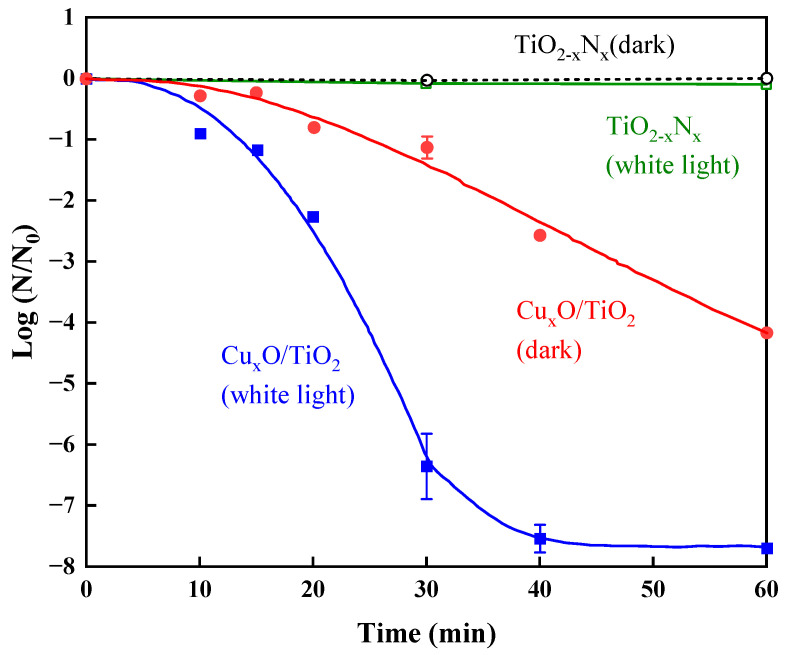
Inactivation efficiency of bacteriophage Qβ by Cu_x_O/TiO and TiO_2−x_N_x_ samples under white light and dark conditions (using white fluorescent bulbs with UV-blocking film for light exposure). Reproduced from [45], Catalysts, with permission from MDPI Press 2020.

Copper oxide nanoclusters (Cu_x_O) are a p-type semiconductor with high visible-light absorption and remarkable electrical properties [44]. Miyauchi et al. [45] produced Cu_x_O (1 < x < 2) nanoclusters by combining Cu(I) and Cu(II) into and grafting them onto the surface of TiO_2_ to obtain a Cu_x_O/TiO_2_ photocatalyst, which presented high inactivation activity against bacteriophages under visible-light irradiation. The material with a ratio of Cu(I)/Cu(II) of 1.3 showed the best performance in maintaining effective visible-light photocatalytic activity and antiviral activity. As shown in Figure 2, the antiviral activity of Cu_x_O/TiO_2_ under visible light was further improved compared to that under dark conditions, and bacteriophage Qβ was reduced by 7.5log in 40 min. This is because Cu(I) has good antiviral activity under dark conditions, while Cu(II) can accept e^−^ from the valence band of TiO_2_ to form Cu(I) by photo-induced interfacial charge transfer transition. Due to the presence of both Cu(I) and h^+^ in the valence band of TiO_2_, Cu_x_O/TiO_2_ exhibits more efficient antiviral performance under visible-light irradiation.

### 2.3. TiON/PdO

**Figure 3:**
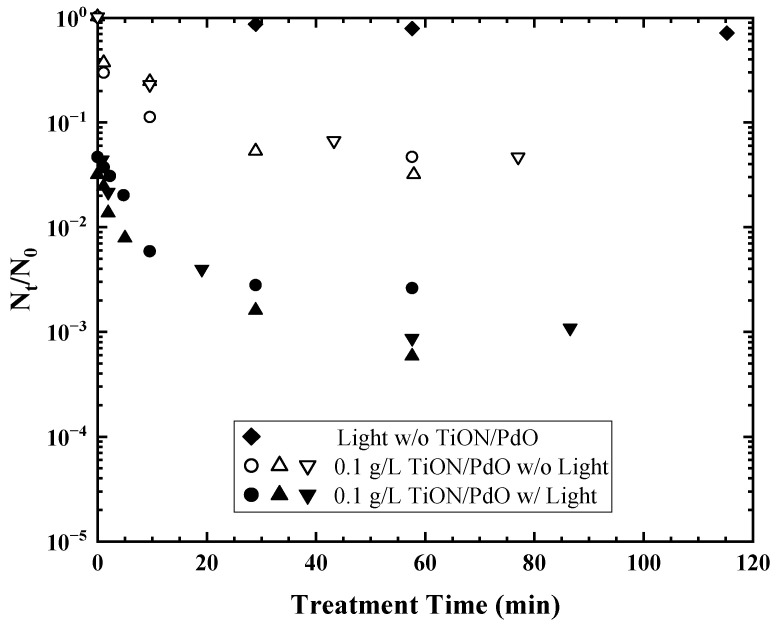
Inactivation of MS2 under different conditions using only visible light (>400 nm) (⧫), TiON/PdO photocatalyst in a dark environment (○Δ∇), and TiON/PdO photocatalyst with visible light (>400 nm) irradiation after corresponding dark control (●▲▼)). Reprinted with permission from [47]. Copyright 2008 American Chemical Society.

It has been reported that the absorption spectrum of TiO_2_ can be extended to the visible-light region by doping nitrogen [46]. Li et al. [47] synthesized a palladium-modified nitrogen-doped titanium dioxide (TiON/PdO) nanoparticle photocatalyst using a sol-gel process, and the composite was further fixed onto a mesoporous activated carbon fiber template for easy recovery of the photocatalyst from water. As is shown in Figure 3, the modification with PdO enhanced the light absorption in the visible range (>400 nm), and the absorption increased with increasing PdO dose when PdO < 2% [48]. The removal rate of bacteriophage MS2 by TiON/PdO reached 94.5–98.2% after 1 h of visible-light irradiation, which was significantly higher than that under dark conditions [47].

### 2.4. ZnO-Based Floating Photocatalyst

**Figure 4:**
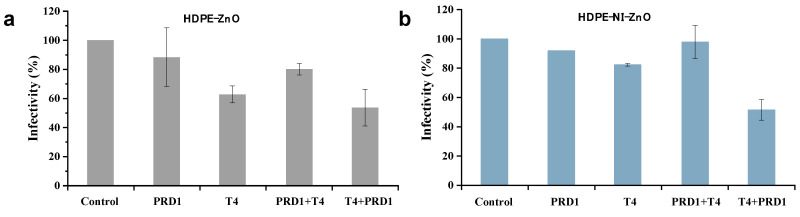
Infectivity of separate PRD1 and T4 phages and their mixture under visible-light irradiation for 1 h using ZnO-HDPE (**a**) without Ni underlayer and (**b**) with Ni underlayer. Reproduced from [53], Materials, with permission from MDPI Press 2022.

ZnO is an environmentally friendly material with a band gap (about 3.2 eV) similar to that of TiO_2_ and similar photocatalytic properties which need to be irradiated by UV to show its activity. Because ZnO is relatively cheap, it is often considered as a substitute for TiO_2_ in photocatalytic applications [49]. Studies have shown that doping Ni can improve the photocatalytic performance of ZnO [50,51]. In order to further enhance the photocatalytic efficiency, a floating composite catalyst has been developed by immobilizing the photocatalyst on a floating substrate to utilize the maximum light source and retain the pollutant near the surface of the photocatalyst. The floating photocatalyst can achieve the maximum oxidation and the best light radiation, greatly improving the generation of free radicals and photocatalytic efficiency [52]. Urbonavicius et al. [53] prepared ZnO-based floating photocatalysts with high-density polyethylene (HDPE) spheres with and without a Ni underlayer as the floatable substrate, respectively, and the ZnO film was deposited onto the HDPE sphere (denoted as ZnO-HDPE). It was found that the use of the ZnO-HDPE photocatalyst could inactivate the phage mixture under visible-light irradiation. Without a Ni underlayer, ZnO-HDPE reduced infectivity by 38% and 47% after irradiation of phage T4 alone and in the mixture with PRD1 (T1 + PRD1) for 1 h, respectively (Figure 4a), while ZnO-HDPE with a Ni underlayer reduced phage infectivity by 18% and nearly 50%, respectively (Figure 4b). Although there was no significant difference in the inactivation effect of the ZnO-HDPE photocatalyst with or without the Ni underlayer on phages, the sensitivity of phage T4 was greatly increased after mixing with phage PRD1.

## 3. Direct Visible Light Response Composite Materials

Graphitic carbon nitride (g-C_3_N_4_) is a π-conjugated semiconductor that can absorb visible light up to 460 nm. It is considered to be a novel material as a visible-light-responsive photocatalyst due to its excellent visible-light response, high chemical stability and non-metal property. However, the band gap (2.7 eV) of g-C_3_N_4_ is relatively large and the recombination rate of photo-excited electrons (e^−^) and holes (h^+^) is very high, which causes the low effective utilization of visible light [54]. Several approaches have been developed to enhance the photocatalytic activity of g-C_3_N_4_.

### 3.1. Graphitic Carbon Nitride (g-C_3_N_4_)

**Figure 5:**
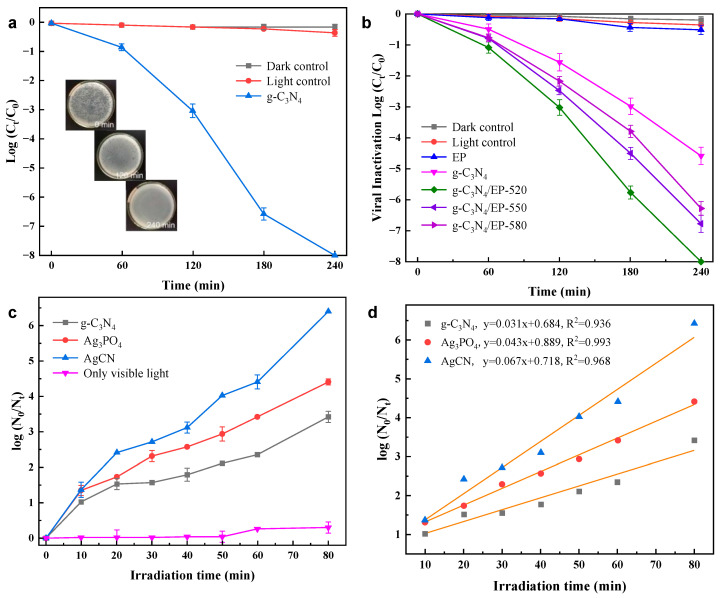
(**a**) Inactivation efficiency against MS2 (∼1 × 10^8^ PFU/mL, 100 mL) by g-C_3_N_4_ under visible-light irradiation under optimal conditions (199.80 mW/cm^2^, 135.40 mg/L and 24.05 °C for light intensity, photocatalyst loading and reaction temperature, respectively). Reproduced from [55], Chemosphere, with permission from Elsevier Ltd. Press 2018. (**b**) The inactivation of MS2 phage by g-C_3_N_4_/EP under visible-light irradiation. Reproduced from [56], Chemosphere, with permission from Elsevier Ltd. Press 2018. (**c**) Inactivation efficiency toward bacteriophage f2 by different materials under visible-light irradiation; (**d**) linear fitting of the kinetic curves of inactivation. Reproduced from [57], Catalysts, with permission from MDPI Press 2018.

Zhang et al. [55] investigated the photocatalytic inactivation of bacteriophage MS2 using a g-C_3_N_4_ photocatalyst by changing the operating parameters, namely light intensity, photocatalyst loading and reaction temperature via a response surface methodology. The study revealed that the order of influence of variables on the light response of g-C_3_N_4_ followed light intensity > photocatalyst loading > reaction temperature. The best performance of photocatalytic virus inactivation was shown under the conditions of light intensity of 199.80 mW/cm^2^, photocatalyst loading of 135.40 mg/L and reaction temperature of 24.05 °C. Under the optimal conditions, the complete photocatalytic inactivation of MS2 at 10^8^ PFU/mL was achieved after 240 min of visible-light irradiation (Figure 5a). The g-C_3_N_4_ was further tested for five consecutive cycles for MS2 inactivation experiments under the optimal conditions, and the results showed the inactivation efficiency remained almost unchanged. It concludes that g-C_3_N_4_ has a stable inactivation performance for photocatalytic MS2 under optimal conditions.

### 3.2. g-C_3_N_4_/EP Floating Composite

Expanded perlite (EP)-supported g-C_3_N_4_ (g-C_3_N_4_/EP) was synthesized and studied for photocatalytic disinfection of MS2 phage [56]. The absorption of visible light by the EP carrier is neglectable, and strong visible-light absorption was observed after loading g-C_3_N_4_. The absorption edge of g-C_3_N_4_/EP shifted to a longer wavelength as the preparation temperature increased. The g-C_3_N_4_/EP-520 (synthesized at 520 °C) presented the best photocatalytic performance. After 240 min of visible-light irradiation at 150 mW/cm^2^, g-C_3_N_4_/EP-520 inactivated 8log MS2 phage (Figure 5b). In general, photocatalytic water disinfection is caused by the redox reaction of photogenerated h^+^ and e^−^ on the surface of the photocatalyst and the production of ROS (e.g., •O_2_^−^ and •OH). However, since e^−^ in the conduction band of g-C_3_N_4_ can reduce O_2_ to •O_2_^−^ and h^+^ in the valence band cannot oxidize OH^−^/H_2_O to •OH, •OH is assumed to play the main role in the inactivation of MS2 using g-C_3_N_4_/EP.

### 3.3. Ag_3_PO_4_/g-C_3_N_4_ Composite Photocatalyst

Ag_3_PO_4_ is a new type of visible-light photocatalyst which has a strong absorption capacity for visible light with a wavelength less than 560 nm. The highly dispersed conduction band of Ag_3_PO_4_ and the sensing effect of PO_4_^3−^ contribute to the separation of photogenerated e and h^+^ [58]. Cheng et al. [57] synthesized Ag_3_PO_4_/g-C_3_N_4_ (AgCN) composites using a hydrothermal method and applied the composites in the inactivation of viruses in water. A strong light absorption edge was shown at 500 nm, which was attributed to the combination of Ag_3_PO_4_ particles. Compared with g-C_3_N_4_, the visible-light absorption capacity of AgCN composites was enhanced. With visible-light irradiation, 6.5log bacteriophage f2 was inactivated by the AgCN composite after 90 min (Figure 5c), and the kinetic rate constant of inactivation of AgCN was about 1.5 times greater than that of g-C_3_N_4_ alone (Figure 5d).

## 4. Carbon Composite Materials

Carbon-based material photocatalysts have received extensive attention in the past decade. Graphene and fullerene (such as C_60_ and C_70_) are commonly used carbon materials in photocatalytic applications at present. Graphene is one of the most studied nano-carbons due to its unique 2D structure and high conductivity. When graphene is combined with semiconductor photocatalysts, it enhances the availability of active sites and augments the adsorption capacity for reagents, consequently bolstering the photocatalytic efficiency of semiconductor materials [59]. Fullerene can strongly absorb UV light and has a moderate absorption of visible light, and is thus expected as a new type of photocatalyst for disinfection. However, fullerenes are usually not used in their original form but are functionalized with different functional groups [44].

### 4.1. Cationic Amine-Functionalized C_60_(HC4)

**Figure 6:**
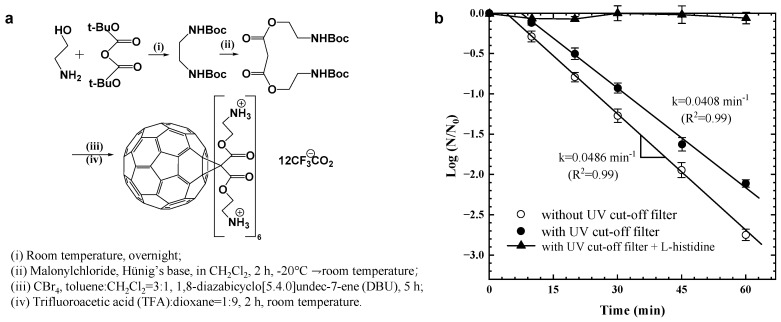
(**a**) Synthesis of cationic hexakis C_60_ derivative (called HC4). (**b**) Inactivation of MS2 phage by HC4 under fluorescent-light irradiation (22 °C, pH = 7.0, [MS2] = 4−6 × 10^4^ pfu/mL, [HC4] = 15 μM, light intensity at 365 nm = 165 μW/cm^2^). Reprinted with permission from [61]. Copyright 2010 American Chemical Society.

C_60_ has extremely low water solubility and can form stable colloidal aggregates in water (nC_60_), resulting in the loss of its original photochemical properties. It was found that the photochemical properties of C_60_ can be preserved by presenting electron transfer when C_60_ bonds with hydrophilic functional groups [60], such as an amine-modified cationic hexakis C_60_ derivative (HC4) synthesized by Cho et al. [61] (Figure 6a), which can produce singlet oxygen (^1^O_2_) under visible-light irradiation for inactivating MS2 phages. The inactivation kinetics were only slightly lower than those of direct sunlight irradiation after using an ultraviolet filter to cut off UV light below 400 nm, indicating that the deactivation was mainly driven by visible-light photocatalysis (Figure 6b).

### 4.2. Immobilized C_60_ Photocatalyst

**Figure 7:**
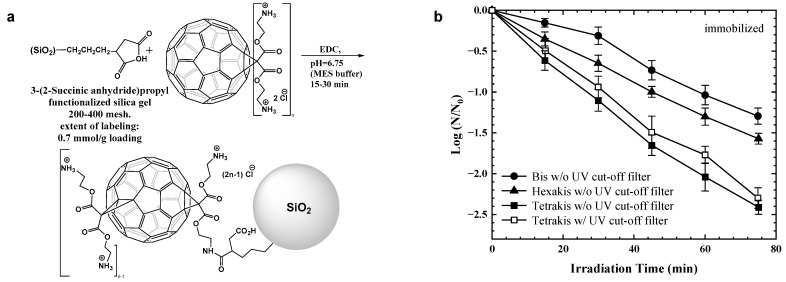
(**a**) Strategy for immobilization of water-soluble C60 amino fullerenes on functionalized silica gel (*n* = 2, 4, and 6). (**b**) MS2 phage inactivation by immobilized bis-, tetrakis-, and hexakis-amino C60/silica under visible light and under commercial fluorescence lamp irradiation. Reprinted with permission from [62]. Copyright 2010 American Chemical Society.

Lee et al. [62] proposed a C_60_ amino fullerene immobilized on surface-functionalized silica gel, amino C_60_/SiO_2_ (Figure 7a). The immobilization improved the photochemical production rate of ^1^O_2_ in the C_60_ amino fullerene solution, and the highest production rate was observed with tetrakis-amino C_60_/SiO_2_, which also exhibited the best inactivation effect on MS2 phages (Figure 7b). Cutting off the light below 400 nm did not decrease the inactivation kinetics significantly, corroborating that the tetrakis-amino C_60_/SiO_2_ inactivation of viruses is mainly induced by visible light. Moor and Kim [63] developed a silica-supported fullerene material (C_60_/SiO_2_) that involved the simple nucleophilic addition of a primary amine across a [6,6] fullerene double bond. It was confirmed that C_60_/SiO_2_ prepared with the direct addition of amine had inactivation effects on MS2 phages under visible-light irradiation. MS2 showed a 2 to 2.5 log inactivation rate after 40 min of visible-light irradiation. Choi et al. [64] prepared a C_60_ amino fullerene–magnetite nanocomposite (C_60_/msu-SiO_2_/mag) by covalently immobilizing C_60_ amino fullerene on functionalized mesoporous mesocellular silica (msu-SiO_2_) with embedded Fe_3_O_4_ nanoparticles (msu-SiO_2_/mag). This magnetic silica-immobilized amino fullerene material can effectively inactivate MS2 phages by producing ^1^O_2_ under visible-light irradiation.

### 4.3. Immobilized C_70_ Photocatalyst

**Figure 8:**
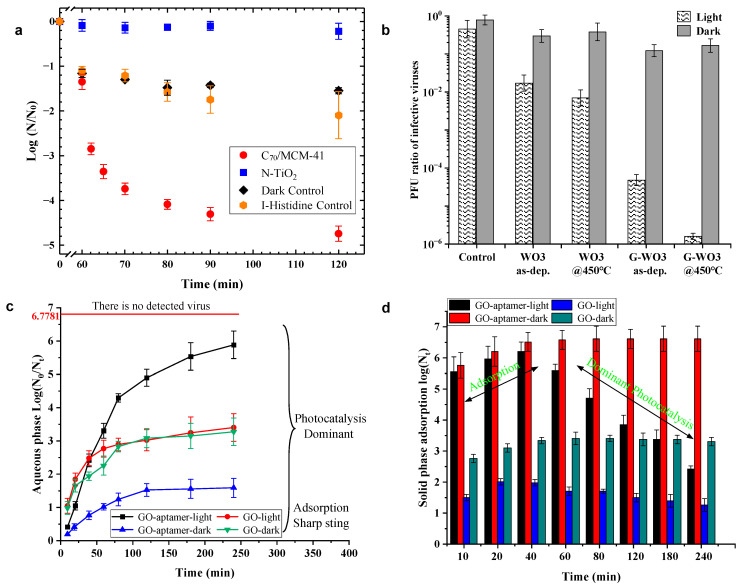
(**a**) MS2 inactivation kinetics of C_70_/MCM-41 under visible-light irradiation. Reprinted with permission from [65]. Copyright 2015 American Chemical Society. (**b**) PFU ratio of the infective bacteriophage MS2 viruses remaining on the surfaces of the various thin films under visible-light irradiation and in the dark for 3 h. (Note: The control sample is a glass substrate without any coating). Reprinted with permission from [67]. Copyright 2012 American Chemical Society. Inactivation of MS2 by GO–aptamer nanosheets and GO nanosheets under visible-light or dark conditions (**c**) aqueous phase and (**d**) solid. Reproduced from [68], Carbon, with permission from Elsevier Ltd. Press 2012.

C_70_ has similar visible-light absorption characteristics to C_60_, but with a stronger absorption. C_70_ also has a large ^1^O_2_ quantum yield, making it an effective photosensitizer. An amine-functionalized silica support (MCM-41)-immobilized C_70_, C_70_/MCM-41, was developed by Moor et al. [65]. Compared with the previous C_60_ and its supported derivative materials, the visible-light activity of MCM-41-immobilized C_70_ showed a significant improvement. About 3log of virus can be inactivated after 60 min of visible-light irradiation by C_70_/MCM-41 and 5log after 120 min irradiation (Figure 8a).

### 4.4. Graphene–Tungsten Oxide Composite (G-WO_3_)

A composite material composed of semiconductor material and graphene can act as an electron acceptor to hinder the recombination of electron–hole pairs, thus improving the photocatalytic activity of the composite material. These composites promote the development of new materials for the photocatalytic oxidation process [66]. Akhavan et al. [67] developed a graphene–tungsten oxide composite (denoted as G-WO_3_) by incorporating graphene oxide (GO) into tungsten oxide film following film heat treatment. The study shows that G-WO_3_ reduces 99.999% of phage MS2 after 3 h of visible-light irradiation (Figure 8b), demonstrating an excellent visible-light response to inactivated phage MS2.

### 4.5. Graphene Oxide–Aptamer Nanosheet (GO^−^Aptamer Nanosheet)

Graphene oxide (GO) has attracted extensive attention due to its high yield, unique surface chemical characteristics and high biocompatibility [66]. However, GO has few specific binding sites to the target and is easy to aggregate, which affects the photocatalytic efficiency. Aptamer is a single-stranded nucleic acid that can bind chemical molecules, nucleic acids, proteins and even microparticles. Hu et al. [68] obtained GO–aptamer nanosheets by covalently bonding the aptamer to GO; GO–aptamer exhibited an obvious reactivity to destroy proteins, suggesting that aptamer played a critical role in photocatalysis, thus improving the photocatalytic activity of GO. As shown in Figure 8c, the inactivation effect of GO–aptamer nanosheets on viruses is better than that of GO nanosheets. After 250 min of visible-light irradiation, the GO–aptamer nanosheets can remove 99.999% of MS2 phage, which is 5 orders of magnitude higher than the dark reaction group. As presented in Figure 8d, the adsorption reached equilibrium after 40 min in the GO–aptamer dark group, and the survival of viruses was dramatically reduced (3 orders of magnitude) on GO–aptamer from 60 min to 240 min of irradiation, presenting a powerful photocatalysis. Figure 8c,d support the dominance of photocatalysis in the inactivation of viruses, with the adsorption from aptamer enhancing the reaction.

## 5. Photocatalytic Mechanism

**Figure 9:**
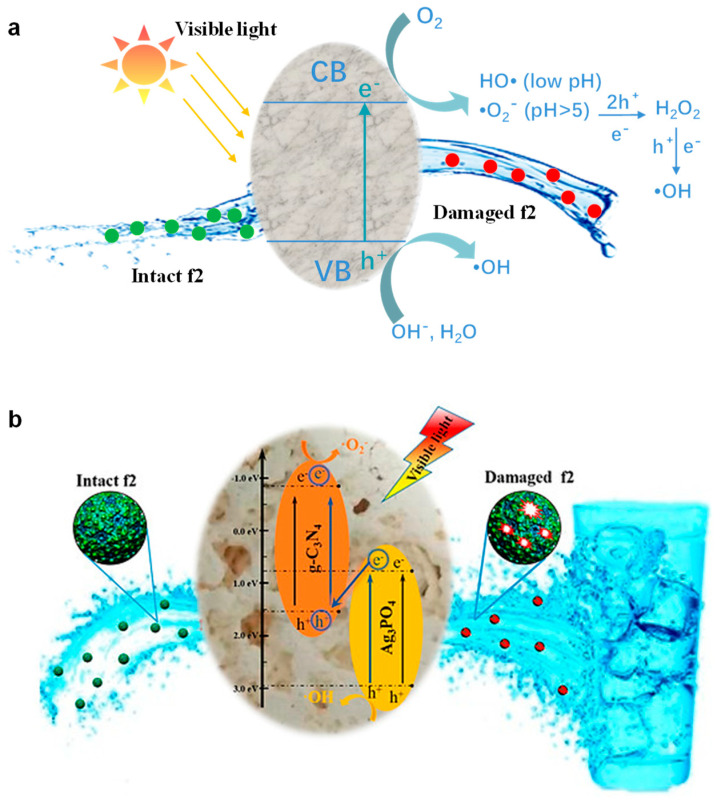
(**a**) Schematic diagram of photocatalytic disinfection mechanism. (**b**) The possible mechanism of photocatalytic inactivation of bacteriophage f2 by the g−C_3_N_4_/Ag_3_PO_4_ photocatalysts under visible−light irradiation [57]. Catalysts, with permission from MDPI Press 2018.

Photocatalytic disinfection mainly relies on the oxidizing free radicals produced to inactivate microorganisms. When a photocatalyst is excited by a photon, electron and hole pairs are produced [20]. Holes have oxidizing properties and can directly oxidize microorganisms in contact with photocatalysts, destroying the structure and function of microorganisms [69]. Holes can also react with H_2_O or OH^−^ (at higher pH) to form •OH, thereby destroying the proteins, nucleic acids, lipids and other components of microorganisms via oxidation reactions, and ultimately leading to the death of microorganisms [70,71]. Photogenerated electrons are reducible but can be captured by dissolved O_2_. During the linking reaction, the trapped electrons produce various oxidizing groups. When the pH value is low, the conduction band electrons meet the energy level requirements of O_2_ reduction to form HO•. At higher pH (<5), O_2_ can be reduced to O_2_^•−^ by conduction band electrons. O_2_^•−^ react with two dissociated hydrogen ions and a photogenerated electron to form H_2_O_2_. H_2_O_2_ can react with a dissociated hydrogen ion and a photogenerated electron to form •OH [72]. A schematic diagram of the photocatalytic mechanism is shown in Figure 9a. According to the above discussion, a possible photocatalytic inactivation mechanism of the g-C_3_N_4_/Ag_3_PO_4_ composite photocatalysts is shown in Figure 9b. Both g-C_3_N_4_ and Ag_3_PO_4_ can be irradiated to generate electron–hole pairs under visible-light irradiation. The photoexcited electrons from the valence bands (VBs) of g-C_3_N_4_ and Ag_3_PO_4_ efficiently transfer to their respective conduction bands (CBs). Moreover, electrons from the CB of Ag_3_PO_4_ can migrate to the VB of g-C_3_N_4_. The electrons stored in the CB of g-C_3_N_4_ react with dissolved oxygen, resulting in the formation of reactive O_2_^•−^. Simultaneously, the holes (h^+^) in the VB of Ag_3_PO_4_ react with water or OH^−^, generating •OH. These highly active radical species, along with photogenerated holes, contribute to the oxidation of bacteriophage f2.

In summary, photocatalytic disinfection leverages the production of oxidizing free radicals, such as holes and hydroxyl radicals, to disrupt the molecular structure of microorganisms. This mechanism provides photocatalytic disinfection with broad-spectrum antimicrobial efficacy, exhibiting relatively low resistance from various microorganisms. Moreover, it addresses concerns about secondary pollution. Consequently, photocatalytic disinfection has diverse applications in water treatment, environmental sanitation and the healthcare sector.

## 6. Summary and Discussions

Considering both removal efficiency and time consumption rate, Cu(II) −TiO_2_, Cu_x_O/TiO_2_ nanocomposites and TiON/PdO are more recommended. Table 1 summarizes the above visible-light photocatalyst materials for virus disinfection, and the following conclusions can be drawn.

(1) Common semiconductor materials (such as TiO_2_ and ZnO) have been used for photocatalytic disinfection in the past few years, which, however, can only be activated by UV. Methods have been developed to extend the light-absorption range of ultraviolet materials to achieve visible-light response, thereby performing photocatalytic disinfection in the visible-light region.

(2) Graphitic carbon nitride can be directly activated by visible light to achieve light response, but the utilization rate of visible light is low. The inactivation efficiency can be improved by changing the parameters of the disinfection process and by using a floating base material or compounding with other visible-light catalytic materials to enhance its photocatalytic effect.

(3) Carbon-based materials are promoted as photocatalysts for water disinfection due to their low cost and no risk of metal contamination. Graphene and fullerene materials can achieve light response in visible regions and have good disinfection properties. The functionalization and immobilization of carbon-based materials can further increase the efficiency of photocatalytic virus inactivation and be applied to practical applications.

## 7. Conclusions and Prospects

This paper reviews the latest developments in the utilization of visible-light composite photocatalysts for phage inactivation, encompassing the synthesis, characteristics, disinfection mechanism and overall efficiency of photocatalysts. Three types of photocatalytic materials have been reviewed. Photocatalysis as a green oxidation technology has opened up a simple and efficient way to inactivate viruses in water. The most widely used photocatalyst is TiO_2_. However, with continuous exploration and study, many novel photocatalyst materials have been developed, and their performance exceeds that of TiO_2_, which shows great potential in photocatalytic disinfection. To date, visible-light composite catalysts have demonstrated exceptional efficacy in virus removal. The ongoing research and development of visible-light catalytic materials remain a prevailing trajectory for the future. Efforts to enhance the efficiency and adaptability of photocatalytic processes are driving the exploration and proposition of novel material compositions. This includes the integration of cutting-edge nanomaterials like organic semiconductors, metal–organic frameworks (MOFs), covalent organic frameworks (COFs) and quantum dots. MOFs possess a remarkably adjustable pore structure, leveraging their role in photocatalysis for adsorption, electron transfer and catalytic reactions. Specifically, photoresponsive MOFs exhibit potential for photocatalytic water splitting to generate hydrogen. Meanwhile, COFs boast exceptional electronic conductivity, predominantly applied in photocatalysis for carbon dioxide reduction and water splitting. Carbon quantum dots exhibit remarkable optoelectronic properties and can synergistically combine with other photosensitive materials, such as TiO_2_ or ZnO, to enhance their photocatalytic activity. However, the widespread practical application of these catalysts encounters obstacles such as high production costs and intricate synthesis methods. Furthermore, most of the photocatalytic materials are produced as powder, leading to a pressing need within the field for the creation of easily recyclable photocatalysts. The evolution of composite photocatalytic materials for disinfection is advancing across various formats such as synthetic sprays, gels, masks and more. The introduction of these composite materials into sprays has proven highly effective in eradicating airborne bacteria and viruses via photocatalytic processes. Gel-based applications provide a convenient and long-lasting solution for personal hygiene in hand-sanitizing gels, ensuring extended disinfection. Incorporating composite photocatalytic materials into mask manufacturing serves to filter airborne microorganisms, curbing the spread of pathogens. Maintaining cost-effectiveness is pivotal for the widespread adoption of these products in large-scale applications. Addressing safety concerns related to potential adverse effects on human health is equally crucial. Rigorous scientific evaluation and stringent quality control protocols are imperative to ensure the reliability of these technologies, providing a dependable means for disease prevention within society. More applicable visible-light composite catalysts need to be further studied and explored in the future.

## Figures and Tables

**Table 1:** Summary of Visible-Light Photocatalytic Materials for Virus Inactivation.

Type of Composites	Photocatalyst	Target Microbe	Initial Concentration (PFU/mL)	Light Source and Wavelength	Light Intensity (mW/cm^2^)	Efficiency	Time (min)	Reference
Modified ultraviolet-response material	Cu-TiO_2_ nanofibers	Bacteriophage f2	1 × 10^5^	300 W Xe lamp	70	−2.5log	240	[40]
	Cu(II)-TiO_2_	Bacteriophage Qβ	1 × 10^8^	Fluorescent lamp	NA	−6.5log	30	[43]
	Cu_x_O/TiO_2_ nanocomposites	Bacteriophage Qβ	1 × 10^8^	Fluorescent lamp	NA	−7.5log	40	[45]
	TiON/PdO	Bacteriophage MS2	3 × 10^8^	1000 W xenon-arc lamp	40	94.5–98.2%	60	[47]
	ZnO-based floating photocatalyst	Phage T4	1 × 10^8^	High power led	65	62–82%	60	[53]
		Phage T4 + Phage PRD1	1 × 10^8^	High power led	65	50–53%	60	[53]
Direct visible-light response materials	g-C_3_N_4_	Bacteriophage MS2	1 × 10^8^	Xe lamp	199.8	−8log	240	[55]
	g-C_3_N_4_/EP	Bacteriophage MS2	1 × 10^8^	300 W Xe lamp	150	−8log	240	[56]
	Ag_3_PO_4_/g-C_3_N_4_	Bacteriophage f2	1 × 10^6^	Xe lamp	300	−6.5log	80	[57]
Carbon materials	Cationic hexakis C60	Bacteriophage MS2	4–6 × 10^4^	Fluorescent lamp	165	−2.1log	60	[61]
	tetrakis-amino C_60_/SiO_2_	Bacteriophage MS2	2 × 10^5^	Fluorescent lamp	165	−2.4log	75	[62]
	C_60_/SiO_2_	Bacteriophage MS2	5 × 10^4^	Fluorescent lamp	NA	−2–−2.5log	40	[63]
	C_60_ Amino fullerene-magnetite	Bacteriophage MS2	1 × 10^6^	300 W Xe lamp	NA	−2.7log	75	[64]
	C_70_/SiO_2_	Bacteriophage MS2	3 × 10^8^	Fluorescent lamp	4.23	−4.35log	90	[65]
	G-WO_3_	Bacteriophage MS2	2 × 10^6^	Mercury lamp	110	−5.9log	180	[67]
	GO-aptamer nanosheet	Bacteriophage MS2	6 × 10^6^	300 W tungsten lamp	NA	−5.9log	240	[68]

## Data Availability

The data are openly available without restrictions.
